# The Analysis and Suppressing of Non-Uniformity in a High-Speed Spike-Based Image Sensor

**DOI:** 10.3390/s18124232

**Published:** 2018-12-02

**Authors:** Jing Gao, Yanzhao Wang, Kaiming Nie, Zhiyuan Gao, Jiangtao Xu

**Affiliations:** 1 School of Microelectronics, Tianjin University and Tianjin Key Laboratory of Imaging and Sensing Microelectronic Technology, No. 92 Weijin Road, Nankai District, Tianjin 300072, China; gaojing@tju.edu.cn (J.G.); wyztjdx2012@foxmail.com (Y.W.); xujiangtao@tju.edu.cn (J.X.); 2 School of Computers, Guangdong University of Technology, Guangzhou 510006, China; gaozhiyuan@tju.edu.cn

**Keywords:** interspike time interval, high-speed scanning sequential format, non-uniformity, coefficient matrix, calibration algorithm

## Abstract

In this paper, the non-ideal factors, which include spatial noise and temporal noise, are analyzed and suppressed in the high-speed spike-based image sensor, which combines the high-speed scanning sequential format with the method that uses the interspike time interval to indicate the scene information. In this imager, spatial noise contains device mismatch, which results in photo response non-uniformity (PRNU) and the non-uniformity of dark current. By multiplying the measured coefficient matrix the photo response non-uniformity is suppressed, and the non-uniformity of dark current is suppressed by correcting the interspike time interval based on the time interval of dark current. The temporal noise is composed of the shot noise and thermal noise. This kind of noise can be eliminated when using the spike frequency to restore the image. The experimental results show that, based on the spike frequency method, the standard deviation of the image decreases from 18.4792 to 0.5683 in the uniform bright light by using the calibration algorithm. While in the relatively uniform dark condition, the standard deviation decreases from 1.5812 to 0.4516. Based on interspike time interval method, because of time mismatch and temporal noise, the standard deviation of the image changes from 27.4252 to 27.4977 in the uniform bright light by using the calibration algorithm. While in the uniform dark condition, the standard deviation decreases from 2.361 to 0.3678.

## 1. Introduction

High-speed target recognition, as a branch of visual technology, is to identify objects in the field, for instance, for vehicle tracking [1]. In recent years, with the deepening of research on target recognition, how to let machine capture high-speed moving objects has attracted more and more attention. To meet these needs, high-speed image sensors have been developed rapidly. They can be classified into two types. One is the continuous image sensor [2,3,4]. Its frame rate is relatively low. In some areas, where the frame rate needs to reach kfps~Mfps, this imager will no longer be applicable. Another one is the burst image sensor. The frame rate of this kind of image sensor can reach extremely high [5,6], which means the high-speed demand can be satisfied, yet the image sensor’s ability of continuous perception is sacrificed. The number of consecutive frames is no more than 100. Besides, an extremely high frame rate will bring huge power consumption and large data volume, which becomes the bottleneck for the development of this image sensor. In summary, for the high-speed sensor, the focus of research has been to increase the speed of detection, while making sure that the output data rate and power consumption remain low.

In the past decade, in view of the deepening of biological vision research, biomimetic silicon retinal has been developed rapidly based on silicon technology. A silicon retina-based image sensor named the Dynamic Visual Sensor (DVS) [7,8] has attracted more attention in recent years. In this image sensor, the change of light intensity is converted into a series of spikes or events. Each pixel just detects the variation of the light and no spike will be generated if the light intensity is constant. Therefore, plenty of static background or redundant information that the high-speed image senor puts out is eliminated by the pixel when it receives the scene information. In this way, the amount of data and power consumption are greatly reduced. In addition, a new asynchronous communication pattern called Address Event Representation (AER) [9,10] is adapted to this image sensor. This mode of communication eliminates the concept of frame rate by using handshake protocol as a bridge of communication. In this way, the image sensor has the ability to capture fast moving objects continuously with an extremely low data rate. The sensor in reference [11] is able to capture objects rotating at 10,000 revolutions per second. However, as arbiter is employed to judge the conflicts when multiple pixel issue output requests at the same time, this communication protocol still has a drawback where the event rate has a ceiling because of the limitation of the output bandwidth. In reference [12], the max bandwidth is 50,000,000 events per second (eps) with the imager’s resolution of 240 × 180. With violent changes in light intensity or with a larger size of the pixel array, the max data bandwidth is easy to reach. In the meanwhile, a large number of pixels are blind and wait the readout command, which will miss subsequent scenes.

**Figure 1:**
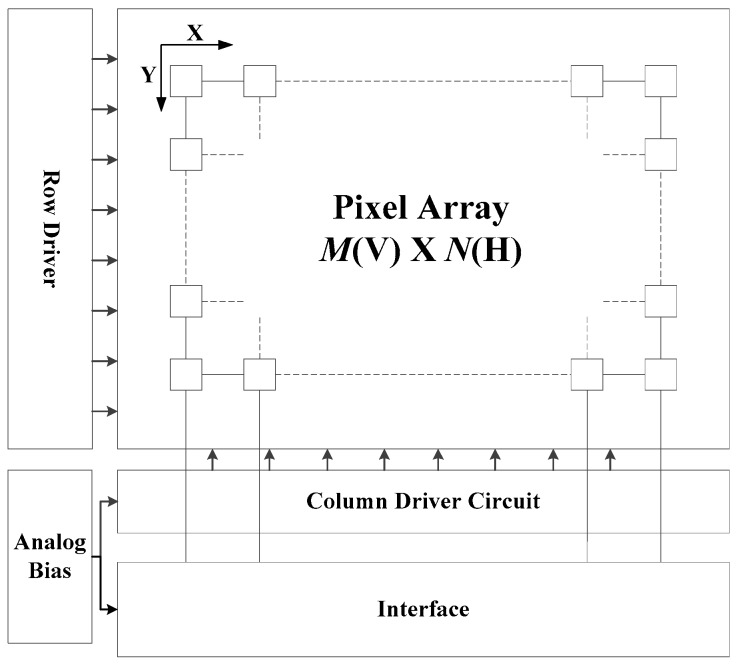
The structure of the spike-based image sensor. *M* is the row number and *N* is column number of the pixel array.

Unlike the DVS, another way that uses the interspike time interval to represent the light intensity has been reported in [9,13]. The image sensor in this paper uses a traditional pulse frequency modulation (PFM) image sensor to generate spikes, and eliminates the multi-bit memory, which can effectively improve the speed detection ability. By converting the light information into two adjacent spikes, plenty of data can be reduced. Besides, the high-speed scanning sequential format means the image sensor has the ability to capture the high-speed moving objects even in a complex scene. Based on the above two considerations, an imager with the pixel structure combining with the interspike time interval representation and synchronous scan readout is shown in Figure 1.

In this paper, the mechanism and influence of non-ideal factors of a spike-based image sensor are discussed. As the pixel continuously perceives the scene information in the time domain, it is impossible to eliminate the influence of photo response non-uniformity (PRNU) by the time domain correlation double sampling method [14,15]. However, based on characteristics of PRNU, a coefficient matrix obtained by the measurement is used to suppress its effect. The non-uniformity of dark current will introduce a coefficient, which is related to the light intensity, and it can be corrected by means of the measured time interval in the dark. The temporal noise is distributed disorderly in the time domain, but its influence can be eliminated by averaging in imaging with sacrificing the high-speed detection capability of the imager. From the experimental results, the non-uniformity in the image caused by non-ideal factors is well suppressed in the uniform light and the scene graph test by using the spike frequency to restore the image. However, when using the interspike time interval to restore the image, the correction effect performs poorly in the bright portion, and effectively in the dark portion. 

This paper is organized as follows: The working principle and detailed modules of the spike-based pixel structure are described in Section 2. In Section 3, the non-ideal factors introduced by the spatial noise and temporal noise and their impacts on the system are discussed. In addition, different correction methods are proposed for corresponding the spatial noise. Experimental results are given in Section 4, and Section 5 gives a conclusion on the calibration algorithm.

## 2. Pixel Architecture

### 2.1. Spike-Based Pixel Structure and Working Principle

**Figure 2:**
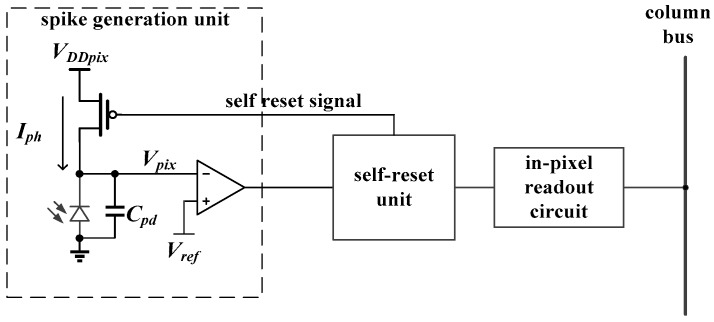

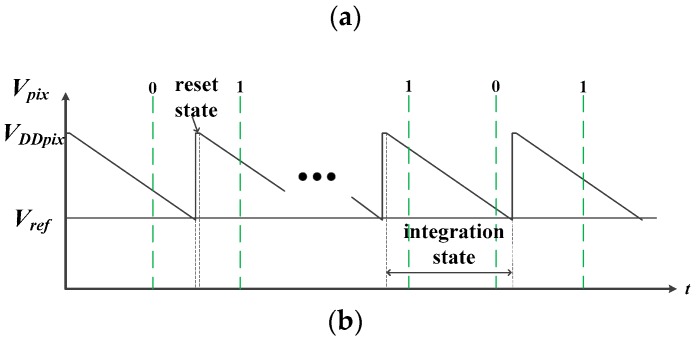
(**a**) The pixel structure and (**b**) working principle. *V_DDpix_* is the supply voltage of the photodiode and *C_pd_* is the capacitance of the photodiode.

Figure 2 shows the pixel structure and its working principle. Figure 2a shows the pixel consists of three parts: spike generation unit, self-reset unit, and in-pixel readout circuit. Figure 2b illustrates the operating principle with three states: integration, reset, and readout. In the integration state, the photodiode in the spike generation unit is employed to convert the light current, *I_ph_*, into the voltage generated by the photodiode, *V_pix_*. The *V_pix_* node voltage will drop with the collection of photoelectrons. When its value reaches the reference voltage, *V_ref_*, the comparator turns over. Once the self-reset unit detects the flip signal, the pixel will enter the reset state. After temporary reset time, a low-level spike, which is used to reset the photodiode, will be generated in the self-reset unit and a new integration stage is resumed. The spike will be stored in the in-pixel readout circuit in the meanwhile. In the readout state, the spike is sequentially scanned and transmitted to the column bus. The dashed line in Figure 2b is the readout state. There will be two kinds of results: “0” and “1”, where “0” means that there is no trigger spike completed within a readout cycle and “1” means that the trigger is completed. Once the information has been readout by the high-speed scanning sequential format, the in-pixel readout circuit will be cleared to receive the next round of signals. In this way, the pixel can detect the continuous light intensity of the scene without information loss. 

According to the analysis of the working principle, *I_ph_* is inversely proportional to the discharging time, *t*, and the relationship can be expressed as follows:

(1) $$t=\frac{C_{pd}\left(V_{DDpix}-V_{ref}\right)}{I_{ph}}$$

### 2.2. Detailed Pixel Structure and Timing Diagram

**Figure 3:**
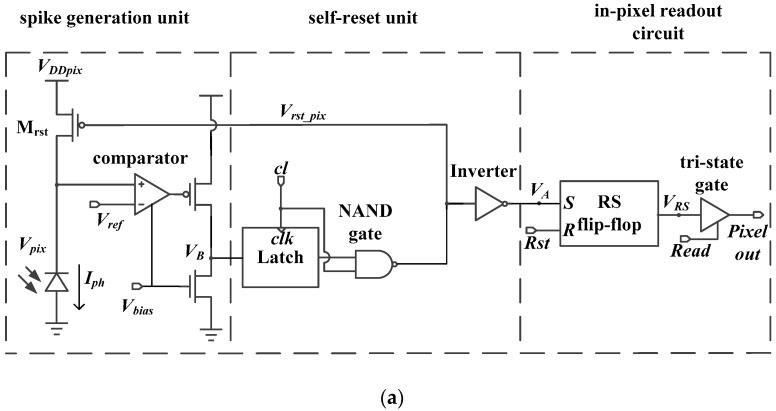

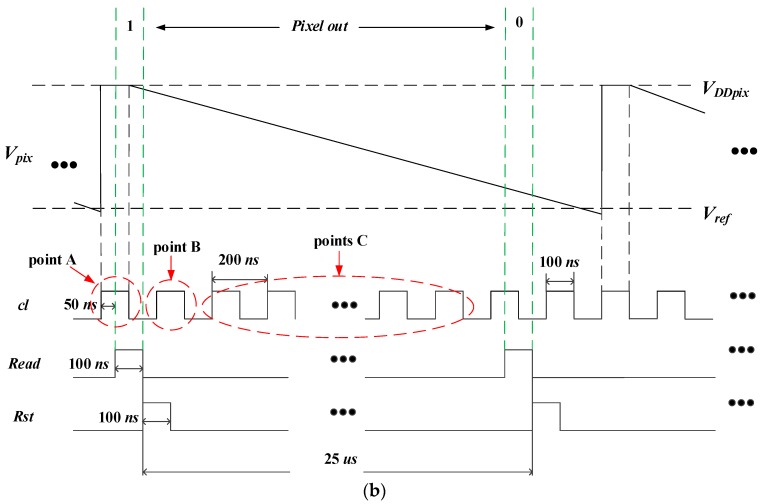
(**a**) Detailed structure and (**b**) timing diagram of the spike-based pixel.

The detailed pixel structure and timing diagram are given in Figure 3. As shown in Figure 3a, the self-reset unit consists of a Latch, a not–and (NAND) gate, and an Inverter. When the Latch control signal, *cl*, is low, Latch is transparent to the input data. Latch will be locked when *cl* is high. The in-pixel readout circuit is composed of a reset-set (RS) flip-flop and a tri-state gate. When the Inverter output signal, *V_A_*, is high, the output of RS flip-flop is set to “1” and will be maintained until the row reset signal, *Rst*, is enabled. For the tri-state gate, once the row scan readout signal, *Read*, is enabled, the data stored in the RS flip-flop will be transmitted to the column bus. Figure 3b shows the timing diagram, where the three signals are all shared by the pixels of one row. The array has 250 (V) × 400 (H) and the row scanning time is 100 ns, so both the period of signal *Read* (as the frame period, *T_read_*, of the pixel) and signal *Rst* are 25 μs. The period of the Latch control signal *cl* is 200 ns.

### 2.3. Synchronous Operation of Comparator Flipping Signal

**Figure 4:**
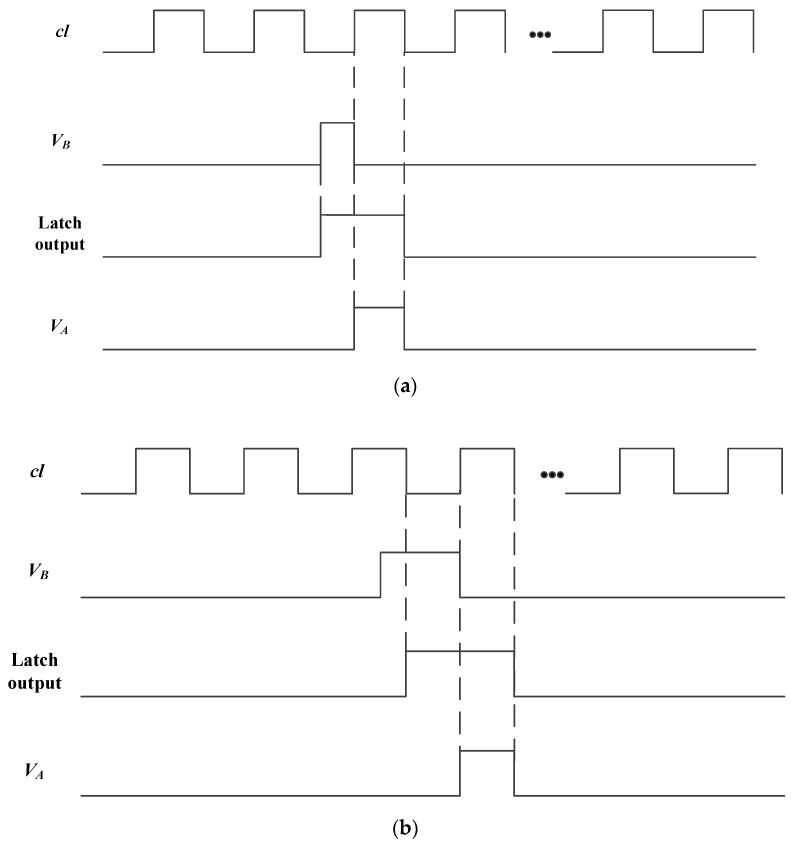
The rising edge of comparator output, *V_B_*, that falls on the (**a**) low level and (**b**) high level of *cl*.

The Latch and NAND Gate play a significant role in synchronizing the output of the comparator with *cl*. Figure 4 shows the two cases of the synchronization process where Figure 4a,b show the rising edge of the comparator output, *V_B_*, that falls on the low level and high level of *cl*, respectively. As we can see in Figure 4, the spike of *V_A_* is just produced during the high level of *cl*. In this way, the flipping signal of the comparator is synchronized to *cl*. When the spike appears at point A of *cl* in Figure 3b, it completes triggering the RS flip-flop. After 50 ns, the output of RS flip-flop, *V_RS_*, is readout by *Read*. For point B, the RS flip-flop is shielded by *Rst* for 50 ns. However, the spike can still trigger the RS trigger through another 50 ns and the information of *V_RS_* will be read out until the next enabled level of *Read* arrives. For the points C, the information of *V_RS_* is readout when the next enable level of *Read* arrives. 

In summary, the timing structure in Figure 3b ensures that the generated spike can be stored and readout. However, the pixel just completes one spike readout process even if two or more spikes are triggered in one readout cycle. The reason is that the RS flip-flop has been set and latched by the time of the first spike comes, and the RS flip-flop will not react to the other spikes. So, the time resolution of the image sensor is 25 μs and the maximum detectable light of the structure can be defined according to *T_read_*.

### 2.4. The Storage and Readout Process of the Generated Spike

**Figure 5:**
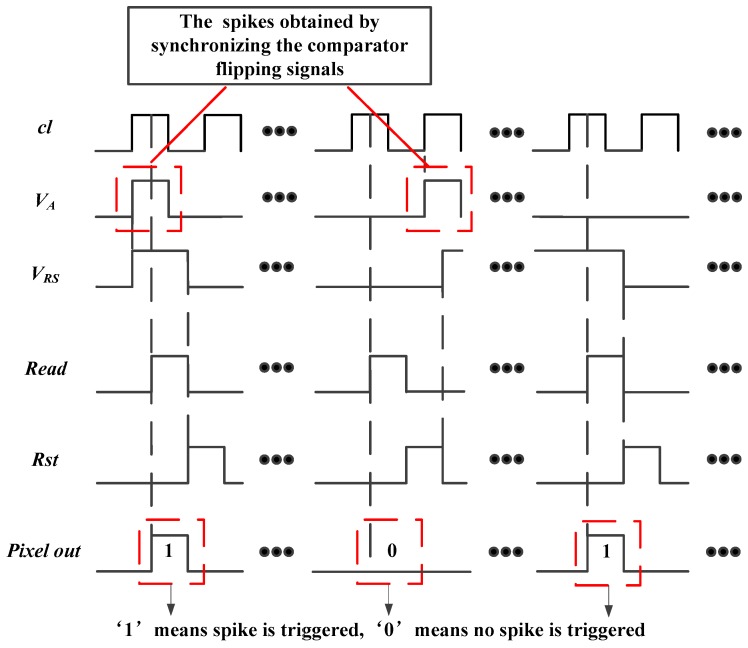
The storage and readout process of a generated spike.

The in-pixel readout circuit is employed to store and output the generated spike. Its two components, the RS flip-flop and tri-state gate, are served as the 1 bit memory and the switch that connects the bus and the pixel, respectively. An example of the storage and readout process of two generated spikes is given in Figure 5. 

As the generated spike has to be stored in pixel to match the synchronous readout process, the spike information observed on the column bus must be the integer multiple of the readout cycle, *T_read_*, which means the spike time interval resolution is 25 μs. Here, we define the time interval between the two ‘1’ observed on the bus column as the interspike time interval, *T*, then we obtain:

(2) $$T=\left[\mathrm{INV}\left(\frac{t}{T_{read}}\right)+1\right]\times T_{read}$$

**Figure 6:**
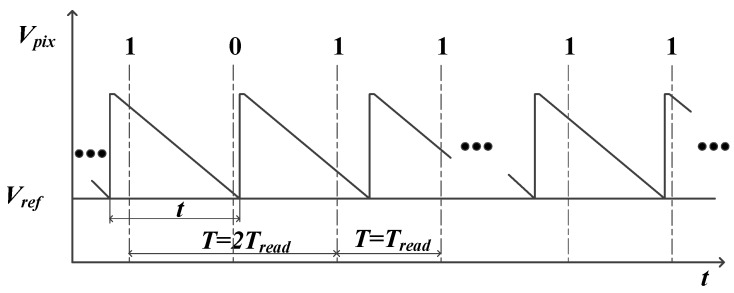
The relationship between the discharging time and interspike time interval.

In (2), INV is the integer function. Here, we can notice that the interspike time interval is not equal to *t*. Besides, the position where the pixel completes the reset operation also affects the interspike time interval. Figure 6 illustrates the operation of a pixel under the bright uniform light intensity. The value of *t* is greater than *T_read_* and less than 2*T_read_*. The first interspike time interval readout from the column is 2*T_read_*, while the second one is *T_read_*. In this way, a time mismatch whose value is uncertain is introduced when using the *T* to replace *t*. However, *t* can be obtained by counting the number of spikes during a period of time, like the PFM image sensor. Yet, it will sacrifice the ability of the imager to detect high speed objects.

### 2.5. Pixel Layout 

**Figure 7:**
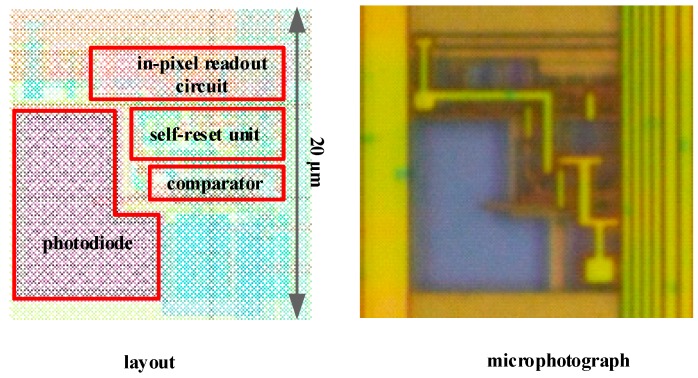
Layout and microphotograph of the spike-based pixel.

The chip has been implemented in a standard 0.11 μm 1-poly 3-metal process. Figure 7 shows the layout and microphotograph of the pixel with the main circuit parts. The square pixel covers 400 μm^2^ of silicon area and its fill factor is 13.75%.

## 3. Noise Analysis and Suppression Methods 

The noise sources of this spike-based image sensor are divided in two kinds: spatial noise, mainly caused by device mismatch and dark current non-uniformity, and noise in the time domain, shot, and thermal noise. This article will introduce their sources and impact on *t*.

### 3.1. Analysis of Spatial Noise 

#### 3.1.1. Analysis of Photo Response Non-Uniformity

In a physical realization, PRNU will be dominated by transistor mismatch between different pixels. Its main source comes from the mismatch of capacitance at the photodiode node, comparator, and reset transistor (M_rst_) in Figure 3a. For capacitance mismatch, it will introduce a coefficient in (1), which can be expressed as:

(3) $$t^{\prime }=\frac{(C_{pd}+\Delta C_{pd})\left(V_{DDpix}-V_{ref}\right)}{I_{ph}}=\lambda t$$

where *t’* is the discharging time with capacitance mismatch, Δ*C_pd_* is the relative deviation value of capacitance, and *λ* is the coefficient introduced by capacitance mismatch and its value is 1 + Δ*C_pd_*/*C_pd_*. 

**Figure 8:**
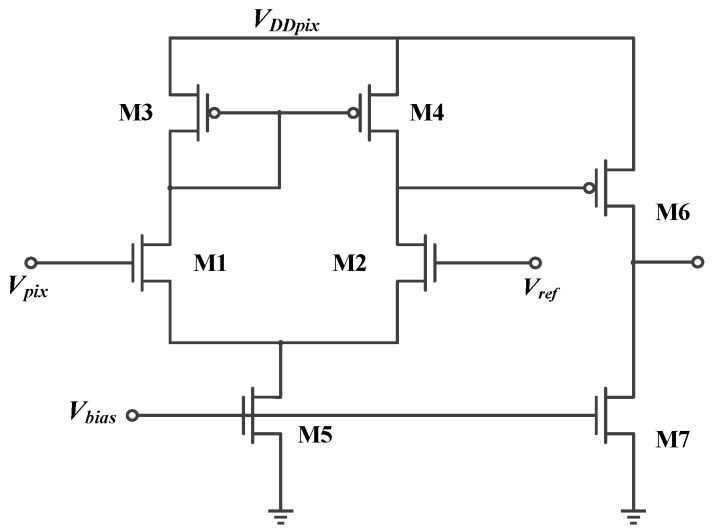
The structure of the in-pixel comparator.

The in-pixel comparator shown in Figure 8 is used to trigger the spike. It must have a small current to reduce the power consumption. Besides, in order to reduce the noise introduced into the pixel, it is necessary to increase the transconductance of M1 and M2 and reduce the transconductance of M3 and M4, which will result in M1 and M2 working in the state of the sub-threshold region and M3 and M4 working in the state of the saturation region. In the subthreshold region, the current formula is given by:

(4) $$I_{D}=\mu C_{d}\frac{W}{L}V_{T}{}^{2}\exp\left(\frac{V_{GS}-V_{TH}}{nV_{T}}\right)\left(1-\exp\frac{-V_{DS}}{V_{T}}\right)$$

where *μ* is the electron mobility, *C_d_* is the gate oxide capacitance per unit area, V_T_ is the thermal voltage, and *n* is the subthreshold slope. Combining (4) with the analysis of noise in [16,17], the offset voltage, *V_OS_*, can be expressed as:

(5) VOS=nVT×Δ(WL)2−Δ(WL)1(WL)1,2−(ΔVTH2−ΔVTH1)+gm3,4gm1,2×[|VGS3,4−VTH3,4|2×Δ(WL)4−Δ(WL)3(WL)3,4+(|ΔVTH3|−|ΔVTH4|)]

where *gm_i_* is the transconductance of the *i*th transistor, and Δ indicates the variation of the corresponding parameters. The value of *V_OS_* is related to the mismatch between M1 and M2 and the mismatch between M3 and M4 in Figure 8. In order to decrease the size of the image sensor, a smaller size of comparator is expected. However, the mismatches will become serious with a small size of the comparator. Then, the value of *V_OS_* will increase according to (5). 

As for the mismatch of the reset transistor, it will lead to the deviation of the reset voltage at the photodiode node, which will also introduce an offset voltage. In this way, the total offset voltage, *V_OS_*,*_tot_*, including the mismatch of comparator and the mismatch of reset transistor will introduce a coefficient in (1), which can be expressed as:

(6) $$t^{\prime \prime }=\frac{C_{pd}\left(V_{DDpix}-V_{ref}+V_{OS,tot}\right)}{I_{ph}}=\phi t$$

where *ϕ* is the introduced coefficient introduced by *V_OS,tot_* and its value is 1 + *V_OS,tot_/*( *V_DDpix_ − V_ref_*), and *t″* is the discharging time with *V_OS,tot_*. 

In summary, considering the effects of capacitance mismatch and offset voltage, this paper proposes a coefficient, *θ*, to represent the total effects in (1), which is given by:

(7) $$t_{mis}=\frac{\left(C_{pd}+\Delta C_{pd}\right)\left(V_{DDpix}-V_{ref}+V_{OS,tot}\right)}{I_{ph}}=\theta t\left(\theta =\lambda \phi \right)$$

where *t_mis_* is the discharging time with the capacitance mismatch and *V_OS,tot_*. The possibility to suppress the effect of PRNU is provided by the coefficient *θ*.

#### 3.1.2. Analysis of Non-Uniformity of Dark Current 

The deviation of the photodiode will also lead to the non-uniformity of dark current, *I_dark_*, which is particularly prominent in low light intensity. It will introduce a coefficient, which is related to light intensity and expressed as:

(8) $$t^{\prime \prime \prime }=\frac{C_{pd}\left(V_{DDpix}-V_{ref}\right)}{I_{ph}+I_{dark}}=\frac{t}{\Psi_{iph}}$$

where *t‴* is the discharging time, including the dark current. *I_dark_* is the dark current, *Ψ_iph_* is the coefficient introduced by the dark current and its value is 1 + *I_dark_*/*I_ph_*.

### 3.2. Analysis of Temporal Noise 

Shot noise, $\bar{V_{shot}}$, is caused by random carriers generated by the Positive-Negative (PN) junction. It is related to the photocurrent, dark current, integration time, and capacitance at the PD node. The randomly generated pair of electrons and holes caused by the photocurrent will result in the shot noise, while the thermal excitation in the depletion region will cause the shot noise of the dark current. It can be expressed as:

(9) $$\bar{V_{shot}}+\bar{V_{rst}}=\sqrt{\frac{q\left(I_{ph}+I_{dark}\right)t}{C_{pd}{}^{2}}+\frac{KT_{a}}{C_{pd}}}$$

where *K* is the Boltzmann constant, and *T_a_* is the absolute temperature. Combining (9) with (1) and the influence of the dark current, the $\bar{V_{shot}}$ can be expressed as:

(10) $$\bar{V_{shot}}=\sqrt{\frac{q\left(V_{DDpix}-V_{ref}\right)}{C_{pd}}}$$

It can be seen from (10) that when *V_DDpix_* is constant, the effect of the shot noise is independent of *I_ph_*, *I_dark_* and *t*, but just related to *V_ref_*. The transient values of $\bar{V_{shot}}$ and $\bar{V_{rst}}$ are unpredictable. However, the influence can be eliminated by time integration. The speed of the image sensor is limited yet.

### 3.3. Methods of Suppressing the Non-Uniformity

#### 3.3.1. Method of Suppressing the Photo Response Non-Uniformity

As the image sensor will work continuously, and the effect introduced by mismatch can be reflected in the form of time integration. A measured coefficient matrix can be obtained to suppress the PRNU. The specific operation flow is described as: the uniform light current, *I_ph_*_1_, is used to irradiate the chip, and then count the spikes per pixel, *N_i,j_*, in the array within a fixed time, *T_total_* (the influence of temporal noise has been eliminated by long-term statistics). The number of spikes of one random pixel, which is considered to have no mismatch, is selected as the reference value, *N*, and the coefficient matrix, *θ_i,j_*, can be obtained through dividing the reference value by the number of spikes of other pixels. 

For the reference pixel, the relationship between the *N* and *T_total_* can be written as:

(11) $$N=\frac{T_{total}I_{ph1}}{C_{pdr}\left(V_{DDpix}-V_{refr}\right)}$$

where *C_pdr_* is the capacitance at photodiode node of reference pixel, and *V_DDpix_* − *V_refr_* is the voltage needed to be released for the reference pixel. For the other pixels, due to the device mismatch (in fact, it is the mismatch between the pixel and reference pixel), we can also obtain:

(12) $$N_{i,j}=\frac{T_{total}I_{ph1}}{C_{pd,i,j}\left(V_{DDpix}-V_{ref,i,j}\right)}=\frac{T_{total}I_{ph1}}{\theta_{i,j}C_{pdr}(V_{DDpix}-V_{refr})}$$

where *C_pd,i,j_* is the capacitance of pixel in (*i*,*j*), and *V_DDpix_* − *V_refr,i,j_* is the voltage needed to be released for the pixel in (*i*,*j*). Through dividing (11) by (12), we obtain the coefficient matrix as:

(13) $$\theta_{i,j}=\frac{N}{N_{i,j}}$$

In this way, the PRNU can be eliminated by dividing the coefficient, *θ_i,j_*.

#### 3.3.2. Method of Suppressing the Non-Uniformity of Dark Current

The dark current has little relationship with the voltage of the photodiode in the image sensor. To facilitate analysis, the value of *I_dark_* is assumed to be constant. Under no light condition, the discharging time, *t_dark_*, can be expressed as:

(14) $$t_{dark}=\frac{C_{pd}\left(V_{DDpix}-V_{ref}\right)}{I_{dark}}$$

Through dividing (14) by (8), the ratio of the dark current to the light current can be obtained as:

(15) $$\frac{I_{dark}}{I_{ph}}=\frac{t^{\prime \prime \prime }}{t_{dark}-t^{\prime \prime \prime }}$$

Then, the value of *Ψ_iph_* is *t_dark_*/(*t_dark_* – *t‴*), and the relationship between *t* and *t‴* can be expressed as:

(16) $$t=\frac{t_{dark}t^{\prime \prime \prime }}{t_{dark}-t^{\prime \prime \prime }}$$

According to (16), it is feasible to correct the discharging time by measuring the matrix of the dark current time interval, *t_dark,i,j_*. 

## 4. Experimental Results

### 4.1. Verification Based on Behavior-Level Model

According to the expressions and working methods in this paper, a behavior-level model of the image sensor with the size of 250 (V) × 400 (H) is established. It can be found in our previous work [18]. Table 1 gives the parameters of the model. The simulation exposure time of the whole experiment is set to 7000 μs. Based on this ideal model, this paper adds non-ideal factors. The mismatch of the *C_pd_* is ± 5% and the influence of *V_OS,tot_* introduced by the mismatch of the comparator and the reset transistor is ± 5%. The range of dark current is between 0.74 pA and 1.82 pA. These non-ideal factor matrices are a random distribution.

#### 4.1.1. Evaluating Indexes

The objective evaluation indexes include the Peak Signal to Noise Ratio (PSNR) and Structural Similarity Index (SSIM). These two indexes are used to evaluate the effectiveness of the calibration algorithm. The indexes are given by:

(17) $$\mathrm{PSNR}\;=\;10\log_{10}\left(\frac{\mathrm{Framesize}\cdot 255^{2}}{\sum_{n=1}^{\mathrm{Framesize}}{\left(R\left(n\right)-F\left(n\right)\right)}^{2}}\right)$$

where Framesize is the size of the picture, and *R* and *F* are the gray value of the reference picture and evaluate picture, respectively.

(18) $$\mathrm{SSIM}\left(x,y\right)\;=\;\frac{\left(2\gamma_{x}\gamma_{y}+c1\right)\left(2\sigma_{x,y}+c2\right)}{\left(\gamma_{x}{}^{2}+\gamma_{y}{}^{2}+c1\right)\left(\sigma_{x}{}^{2}+\sigma_{y}{}^{2}+c2\right)}$$

(19) $$c1\;={\left(k_{1}L\right)}^{2},$$

(20) $$c2\;={\left(k_{2}L\right)}^{2},$$

where *x* and *y* are the pictures that needs to be compared; *γ* is the average value of the picture; *σ* is the standard deviation of the picture, which is given in (21); *L* is the range of the gray value, which is 255; and *k_1_* and *k_2_* are the constant, which are 0.01 and 0.03, respectively.

(21) $$\sigma \;=\;\sqrt{\frac{1}{\mathrm{Framesize}-1}\overset{\mathrm{Framesize}}{\underset{i=1}{\sum }}{\left(x_{i}-\bar{x}\right)}^{2}}$$

where *x_i_* is the gray value of each pixel and $\bar{x}$ is the average of the gray value of all the pixels.

#### 4.1.2. Simulation Results Based on the Spike Frequency

**Figure 9:**
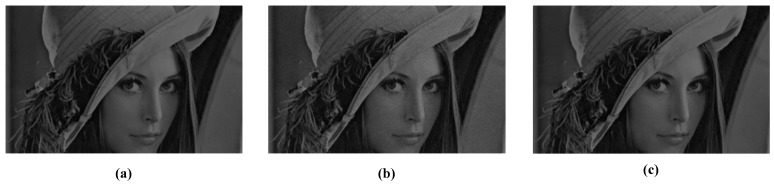
Comparison of images without and with suppression. (**a**) Original image. (**b**) The image with non-ideal factors. (**c**) The image suppressed by the calibration algorithm.

In this paper, the Lena image is used as the input of the sensor. To reduce the effect of synchronous readout on the discharging time, the spike frequency is used to restore the image. The data of 255 read out cycles are added up as the gray value of each pixel. Figure 9 shows the images reconstructed by using this method. Figure 9a shows the image without non-ideal factors, Figure 9b shows the image with non-ideal factors, and Figure 9c shows the image suppressed by the proposed calibration algorithm. Table 2 gives the results of the evaluating indexes. From the results, the non-ideal factors are suppressed. 

#### 4.1.3. Simulation Results Based on the Interspike Time Interval

**Figure 10:**
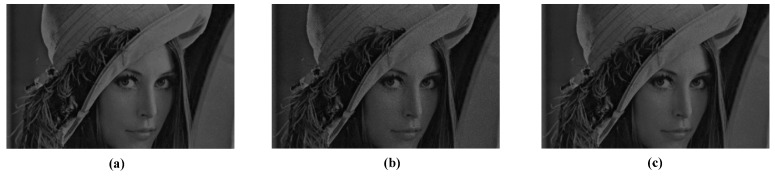
Comparison of images without and with suppression. (**a**) Original image. (**b**) The image with non-ideal factors. (**c**) The image suppressed by the calibration algorithm.

In the high-speed application, the method of restoring images by the spike frequency limits the speed of image sensors, so using the interspike time interval, which can be found in [17], to restore images is desired. Images reconstructed by using the interspike time interval method are shown in Figure 10. Table 3 gives the results of the evaluating indexes. According to the two indicators, the method has an effective effect on suppressing non-ideal factors. 

### 4.2. Verification Based on Camera

**Figure 11:**
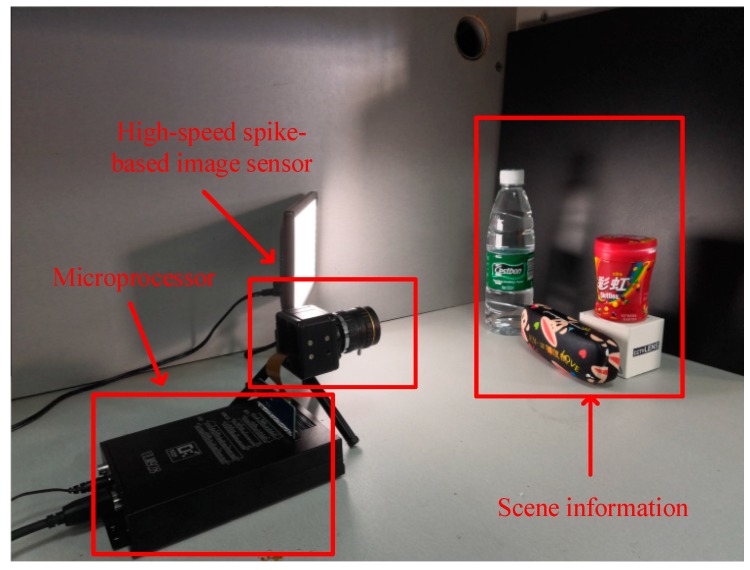
Photograph of the spike-based imaging system.

To verify the effectiveness of the proposed methods, a spike-based Complementary Metal Oxide Semiconductor (CMOS) imaging system is built. As shown in Figure 11, the system consists of the microprocessor, which is used to receive the data and transmit them to the computer, and a high-speed spike-based image sensor, which is used to collect the scene information. Table 4 provides an overview of the imager’s main specifications, important test, and measurement results.

To obtain the coefficient matrix, *θ_i,j_*, introduced by the devices mismatch in (13), the spikes are counted under the light intensity of 400 lux in 40 ms. In this paper, the pixel in the first row and the first column is taken as the reference pixel to obtain the coefficient matrix introduced by PRNU, and matrix *t_dark,i,j_* can be obtained through long time statistics. In this way, the preparation for correcting non-uniformity is ready. 

#### 4.2.1. Evaluation Indexes

In this paper, the experimental results are evaluated by subjective and objective indexes. The subjective indexes include the image visual effect and mean curve [19]. The objective index includes the standard deviation.

#### 4.2.2. Non-uniformity Correction for Images Based on Spike Frequency

**Figure 12:**
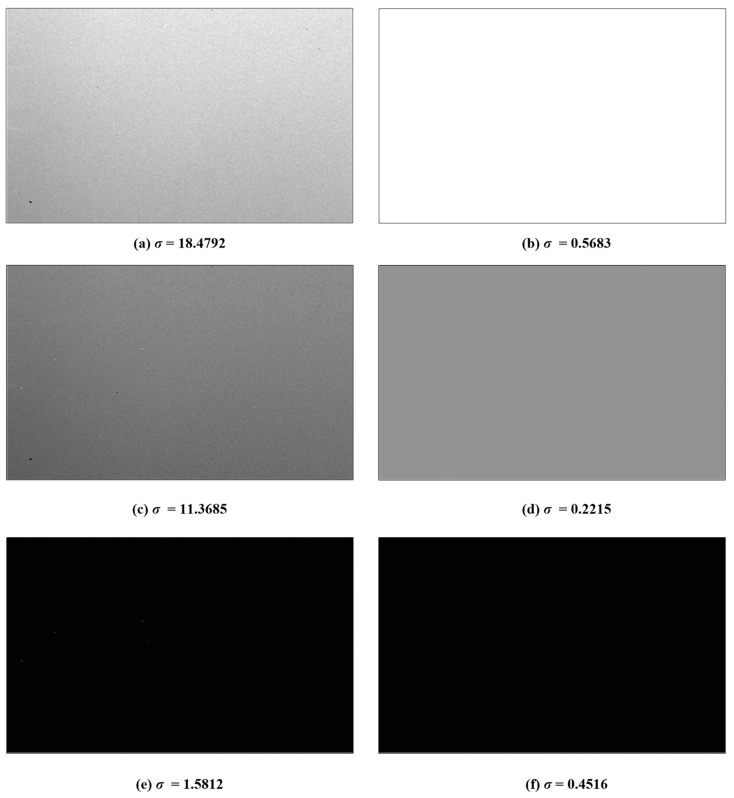
The original images are captured under the (**a**) high, (**c**) moderate, and (**e**) low uniform light intensity. The suppressed results are given corresponding to (**b**) high, (**d**) moderate, and (**f**) low light intensity, respectively. The standard deviation of the image is shown in the bottom.

The original images and the images suppressed by the proposed methods are shown in Figure 12. The images are captured under the different uniform illumination with a resolution of 250 (V) × 400 (H) in Figure 12a,c,e. In these images, the standard deviation, *σ*, is used as an index to evaluate the inconsistency under the uniform illumination. From the results, the higher the intensity of light is, the better the suppressed result will be. 

**Figure 13:**
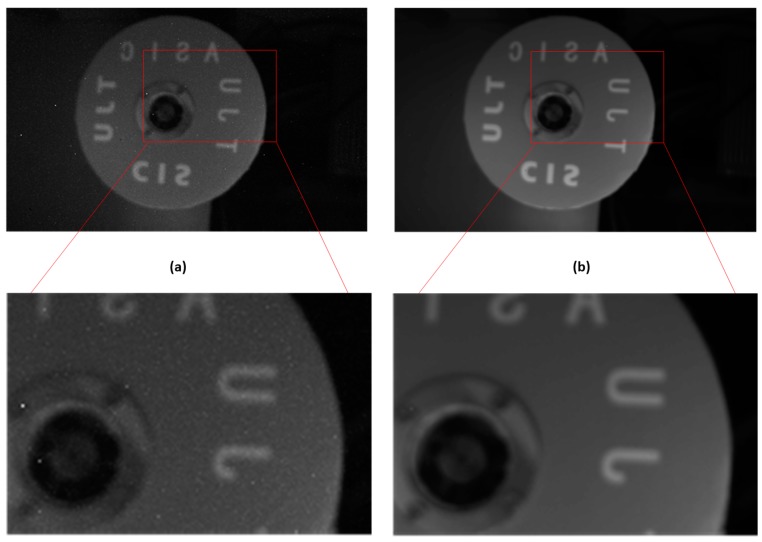
The original scene graph captured by the imager is shown in (**a**), and (**b**) gives the suppressed results.

In order to verify the applicability of the calibration algorithm in any light intensity, a turntable image has been collected. Figure 13a shows the image recovered by the spike frequency, and Figure 13b displays the suppressed image. As shown in these two images, the influences of PRNU and the dark current are effectively suppressed and the image becomes very smooth. 

**Figure 14:**
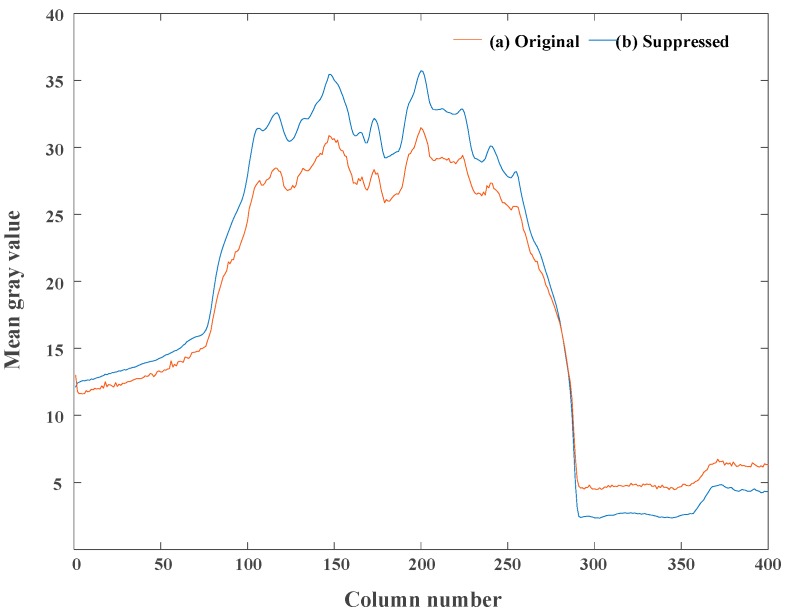
The comparison of column-mean curves of the test images in Figure 13. (**a**) Original curve and (**b**) suppressed curve.

The comparison of the column-mean curve of the image in Figure 13 is shown in Figure 14, where the original curve corresponds to the original image and the suppressed curve corresponds to the image suppressed by the proposed calibration algorithm. After correcting the non-ideal factors, the curve becomes smooth. Besides, the form and trend of the suppressed curve are consistent with the original curve, which demonstrates that the proposed correction algorithm can suppress the non-ideal factors while preserving the original information of the image. 

#### 4.2.3. Non-Uniformity Correction for Images Based on Interspike Time Interval

**Figure 15:**
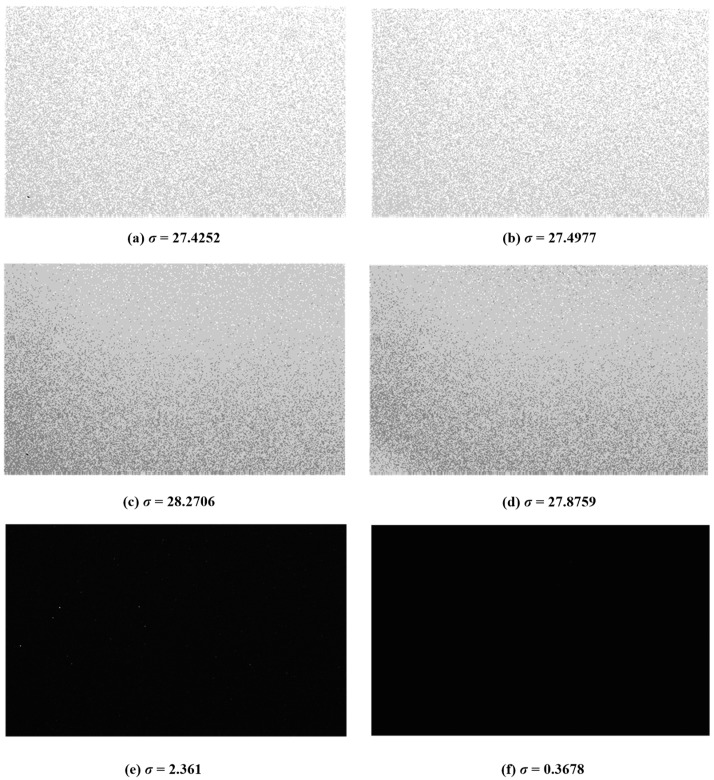
The original images are recovered by the interspike time interval under the (**a**) high, (**c**) moderate, and (**e**) low uniform light intensity. The suppressed results are given corresponding to (**b**) high, (**d**) moderate, and (**f**) low light intensity, respectively.

**Figure 16:**
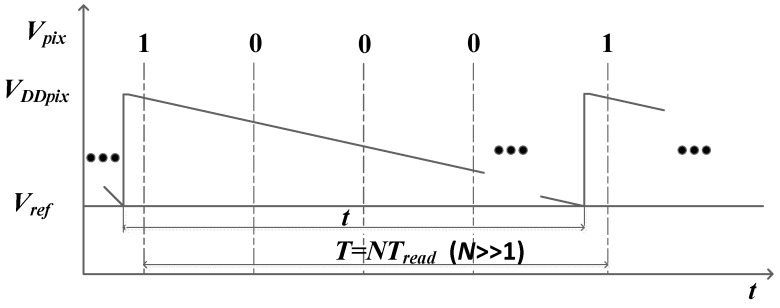
The influence of time mismatch on dark conditions.

**Figure 17:**
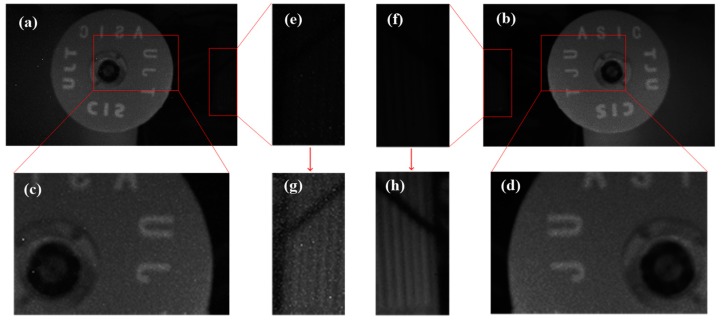
The original image recovered by the interspike time interval in (**a**), and (**b**) gives the suppressed results. The gray values of the dark portion in (**e**) and (**f**) are magnified four times to display in (**g**) and (**h**).

**Figure 18:**
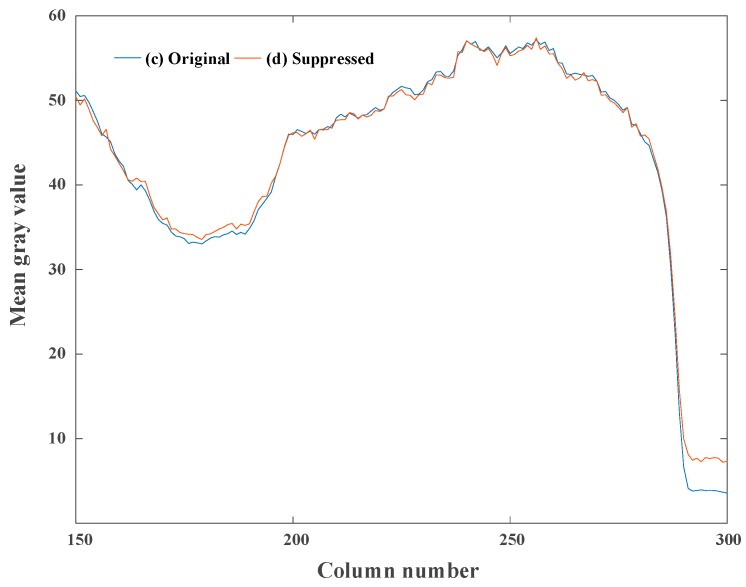
The comparison of column-mean curves of the test images in Figure 17. (**c**) Original curve and (**d**) suppressed curve.

Through using the data collected in Figure 12, the recovered and suppressed images under the different uniform light intensity are shown in Figure 15. The results show that the non-ideal factors are suppressed more effectively in the dark portion compared with the bright portion. The reason is as follows: according to the analysis in Figure 6, the time mismatch is introduced by the synchronous readout. For the bright portion of the image, the influence of the time mismatch and temporal noise on *t* is larger, which leads to a poor correction effect. However, in the dark intensity, the influence of temporal noise is reduced, and *t* is long enough for the time mismatch. Assuming that *t* ≈ *T*, the calibration algorithm will perform effectively. Figure 16 describes the relationship between the *t* and *T* under the dark conditions. Figure 17a gives the original image recovered by the interspike time interval and Figure 17b shows the suppressed image. To observe the correction effect of the image visually, the gray values are strengthened in the dark portion in Figure 17e,f. Figure 18 shows the column-mean curves of the images in Figure 17c,d. According to Figure 18, the smoothness of the suppressed curve is almost unchanged due to the time mismatch in the bright portion.

**Figure 19:**
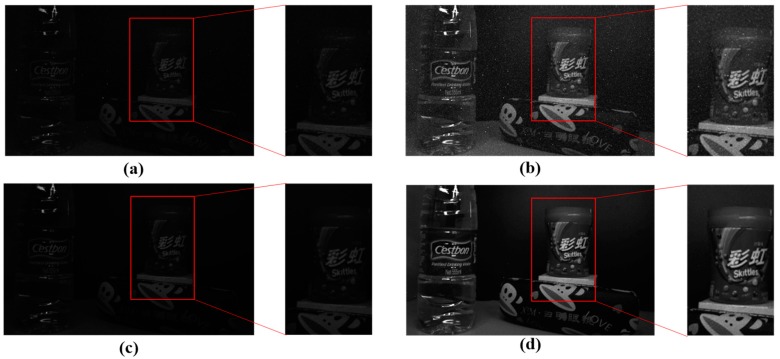
The images restore by the interspike time interval under the dark condition in (**a**). (**c**) Gives the result suppressed by the calibration algorithm. (**b**) and (**d**) are the reinforced results corresponding to (**a**) and (**c**), respectively. The gray values of the whole image are magnified four times to display.

**Figure 20:**
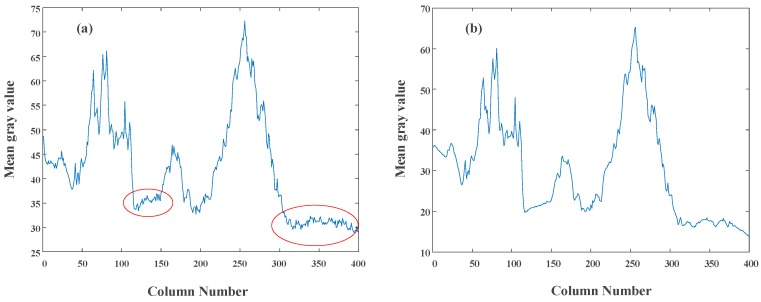
The column-mean curves of (**a**) the original image in Figure 19b and (**b**) suppressed image in Figure 19d.

To verify the influence of time mismatch results from the synchronous readout, an image shown in Figure 19 is captured under the dark condition. To observe the image visually, the gray value is strengthened as well. Figure 20 shows the column-mean curves of the images in Figure 19b,d. The original curve marked with ellipses fluctuates, and the suppressed curve is smooth. The results illustrate the suppression of non-ideal factors under the dark light condition. 

## 5. Conclusions

In the spike-based pixel structure, the non-ideal factors and their influences in the time domain have been discussed clearly in this paper. To suppress their effects, different correction coefficients were proposed. To verify the correctness of the analysis and the calibration algorithm, a system based on this imager of 250 (V) × 400 (H) pixels has been built. Experimental results show that the proposed calibration algorithm suppresses the effect of non-uniformity effectively when the image is recovered by spike frequency. In the uniform light and scene graph test, the effectiveness of the calibration algorithm can be verified. Recovering the image information by the interspike time interval is desired at high speed applications. The correction effect of scene graphs proves that the shorter the discharging time is, the worse the calibration algorithm will be because of the time mismatch and temporal noise.

Further research includes the PRNU elimination in the spike-based image sensor by hardware and photodiode optimization to reduce the effect of the dark current. The author will do further research on this kind of image sensor. 

## Figures and Tables

**Table 1:** Model parameter list.

Parameter	Value
*V_DDpix_*	3 V
*V_ref_*	1 V
*C_pd_*	15 fF
*T_read_*	25 μs
*I_pmax_*	150,000 fA

**Table 2:** Comparison of objective evaluation indexes of the images in Figure 9.

Evaluating Indexes	Image With Non-Ideal Factors	Suppressed Image
PSNR	34.4055	45.3859
SSIM	0.9869	0.9987

PSNR, Peak Signal to Noise Ratio; SSIM, Structural Similarity Index.

**Table 3:** Comparison of objective evaluation indexes of the images in Figure 10.

Evaluating Indexes	Image With Non-Ideal Factors	Suppressed Image
PSNR	37.6171	43.378
SSIM	0.9779	0.9917

**Table 4:** Summary sensor characteristics.

Parameter	Value
Fabrication process	0.11 μm
Supply voltage	3.3 V(analog), 1.5 V(digital)
Chip size	9.96 × 7.1 mm^2^
Array size	250 (V) × 400 (H)
Pixel size	20 μm × 20 μm
Fill factor	13.75%
Fixed pattern noise	2.99% default reference
Random noise	0.75% average interspike time interval *t* is 75 μs, the standard deviation of *t* is 558.48 ns
Frame rate	40 kfps
Time resolution	25 μs
Readout format	LVDS (8 group)/ 500 MHz
Chip data rate	4 Gbps
Power consumption	370 mW
